# New Hampshire Medical Society

**Published:** 1855-09

**Authors:** 


*New Hampshire Medical Society.*—The proceedings of the sixty-fourth anniversary of this ancient and honored society, have been received. From a cursory glance at the report we find much that is highly interesting and valuable. The annual address of Prof. Albert Smith, of Dartmouth College, is a good, clear, logical, and common sense production, exhibiting the author to be not only a deep thinker, but having another commendable quality, that of thinking for himself. We close in with his views on *conservatism* in medicine, indeed there is scarcely any thing in this address to which we could take exception. We make the following extract, which we find at the close of the address, in which will be found the sentiments of all truly good physicians. It is a good mirror in which to find the reflected image of the high souled man and at the same time we will find emphatic expressions of deep condemnation of meanness and knavery. He points out those who bring dishonor upon their profession and says it is "those who are ever trimming their sails to catch the first breath of popular opinion, so as never to be found in minorities, or in any way opposed to their patrons; who, instead of leading, as their qualifications and standing should entitle them to do, are led by the ignorance, credulity and arrogance of their employers—it is these men who bring contempt upon our calling, and induce many of the people to class us on a level with empirics. We shall never fully command respect and confidence until we show ourselves an enlightened, scientific body, actuated by principle and justice, and also as self-possessed and self-relying men, ready for any emergency that can be surmounted by any human means. We rejoice to believe that this improvement is in ample progress, and that a great portion of our profession at this time are well educated, growing men; men who have never, and never will bow to unworthy acts or management for business; who would rather be poor than mean, or starve than be knavishly artful—men who, in the words of Shakspeare, will never

"Bend the pliant hinges of the knee,
That thrift may follow fawning."

While mindful of our obligations to the profession, let us zealously cherish and remember our duties to ourselves. We should be men, as well as physicians, of the highest and noblest character; Christians as well as philosophers; in truth, we cannot be true philosophers unless we are also true Christians."

Such are the sentiments of a man distinguished for the noble qualities of head and heart, and such too are the sentiments of every high minded, honest, and honorable man in the profession.

There should be no conservatism which would justify us in looking indifferently upon the acts of those whose conduct tends to degrade us all. Those who find apologies for such are too loose themselves in the estimate they may make of the character of medical men.

Dr. McFarland pronounced an oration on the poetry of the medical profession. A singular subject and yet well treated. This is followed by a dissertation by Dr. Mason on the chemical changes which take place in the body while in a state of disease.

This society was established in 1791 and enrols among its members some of the most distinguished men of this and other countries, among whom are Jenner, Rush, Wistar, Physick, Barton, Dorsey, Warren, Hosack, Mott, the Becks, and Mussy, with very many others equally distinguished. The proceedings show that Dr. Mussy was present and participated.

